# Tools used for evaluation of Brazilian children's quality of life

**DOI:** 10.1590/0103-0582201432214313

**Published:** 2014-06

**Authors:** João Gabriel S. Souza, Marcela Antunes Pamponet, Tamirys Caroline S. Souza, Alessandra Ribeiro Pereira, Andrey George S. Souza, Andréa Maria E. de B. L. Martins

**Affiliations:** 1 Funorte, Montes Claros, MG, Brasil; 2 Universidade de Aquino Bolivia (Udabol), Santa Cruz de La Sierra, Bolívia; 3 Universidade Estadual de Montes Claros (Unimontes), Montes Claros, MG, Brasil

**Keywords:** quality of life, child, questionnaires

## Abstract

**OBJECTIVE:**

To review the available tools to evaluate children's quality of life validated for Brazilian language and culture.

**DATA SOURCES:**

Search of scientific articles in Medline, Lilacs and SciELO databases using the combination of descriptors "quality of life", "child" and "questionnaires" in Portuguese and English.

**DATA SYNTHESIS:**

Among the tools designed to assess children's quality of life validated for the Brazilian language and culture, the *Auto questionnaire Qualité de Vie Enfant Imagé* (AUQEI), the Child Health Questionnaire - Parent Form 50 (CHQ-PF50), the Pediatric Quality of Life Inventory (PedsQL^(tm))^ version 4.0 and the Kidscreen-52 are highlighted. Some tools do not include all range of ages and some lack domains that are currently considered relevant in the context of childhood, such as bullying. Moreover, due to the cultural diversity of Brazil, it may be necessary to adapt some instruments or to validate other tools.

**CONCLUSIONS:**

There are validated instruments to evaluate children's quality of life in Brazil. However, the validation or the adaptation of other international tools have to be considered in order to overcome current deficiencies.

## Abstract (es)

**OBJETIVO:**

Realizar una revisión de la literatura sobre instrumentos utilizados en la evaluación de la calidad de vida de niños, validados para el Portugués y para la cultura brasileña.

**FUENTES DE DATOS:**

Se investigaron artículos científicos en los portales Medline, Lilacs y SciELO, utilizando la combinación de descriptores "calidad de vida", "niño" y "cuestionarios", además de su versión en inglés. La búsqueda bibliográfica no se limitó a un periodo específico.

**SÍNTESIS DE LOS DATOS:**

Entre los instrumentos creados para evaluar la calidad de vida en niños y que fueron validados para el Portugués y para la cultura brasileña, se destacan el Autoquestionnaire Qualité de Vie Enfant Imagé (AUQEI), el Child Health Questionnaire - Parent Form 50 (CHQ-PF50), el Pediatric Quality of Life Inventory (PedsQL(tm)) version 4.0 y el Kidscreen-52. Se subraya que algunos instrumentos no contemplan todas las edades de la infancia o no poseen dominios considerados relevantes actualmente en el contexto de la infancia, como el bullying. Además, debido a la densidad cultural de Brasil, puede que sean necesarias adaptaciones de los instrumentos existentes o validación de otros.

**CONCLUSIONES:**

Se constata la existencia de instrumentos validados en Brasil pasibles de utilizarse para verificar la calidad de vida de niños. Sin embargo, se considera la necesidad de crear o validar otros instrumentos internacionales para suplir las necesidades existentes.

## Introduction

The World Health Organization (WHO) defined quality of life as "the individual's perception of his/her position in life in the context of culture and value systems in which he/she lives and in relation to his/her goals, expectations, standards, and concerns"^(^ 1 ^)^. This definition emphasizes the multidimensional nature of quality of life that encompasses different aspects, including physical, functional, besides psychological and social well-being^(^ 2 ^,^ 3 ^)^. There is a growing recognition that the quality of life refers to something much broader than health^(^ 4 ^)^. The evaluation of quality of life requires that health professionals not only evaluate biological issues related to the disease, but also consider a psychosocial approach. Consequently, in 1990, it was consolidated the idea that instruments measuring quality of life should consider the perspective of the people and not be restricted to the perspective of health professionals and researchers^(^ 5 ^)^. Previous studies aimed at evaluating quality of life were mostly focused on adults or the elderly^(^ 6 ^,^ 7 ^)^, stressing, however, the need for assessing the quality of life in children^(^ 8 ^,^ 9 ^)^.

The evaluation of quality of life in children was initially held through the perception of parents, considering that children were unable to perform this task. However, further clarification allowed verifying the ability of this population to assess and understand issues related to their lives^(^ 10 ^)^, even though parents may have a different perception of their children^(^ 11 ^)^. Therefore, the inclusion of children as subjects of research depends on their ability, respecting their limits in the complex process related to quality of life and health. It should also be taken into consideration the skill and creativity of the researcher to adapt instruments that become interesting and mobilize children to socialize their experiences^(^ 12 ^)^. Thus, some instruments have been developed with the purpose and possibility of being used to assess quality of life in children^(^ 13 ^-^ 15 ^)^.

In this context, the aim of this study was to review the literature about instruments validated to Portuguese and to the Brazilian culture in order to assess quality of life in children. We researched scientific articles on the Medline, Lilacs, and SciELO databases, using the combination of descriptors "quality of life," "child," and "questionnaires," in English and Portuguese. After the combination of the descriptors and the application of existing filters in databases, such as studies on children, validation studies, and studies conducted in Brazil, 637 scientific studies were found. From reading the abstracts, we excluded studies that did not address the validation of instruments and that aimed to evaluate the quality of life related to a specific health condition or disease. Therefore, we included four studies that validated generic quality of life instruments in children for the Portuguese and Brazilian culture. The literature search was not limited to a specific period. 

## Quality of life Instruments

Questionnaires are instruments that are widely used for scientific research and may be useful, among other things, to estimate the need for treatment, investigate the determinants of the health-disease process, evaluate health service^(^ 16 ^)^, and quality of life. The creation of instruments to assess quality of life that are psychometrically valid presents considerable difficulty due to their subjective characteristics, being influenced by cultural and temporal aspects. Furthermore, it should be considered the multidimensional characteristic of quality of life, that relates to the environment on aspects such as physical, psychological, social relations, and personal beliefs, taking into account that the object of evaluation is the perception of the respondent/patient^(^ 17 ^)^.

In general, indicators of quality of life are associated with health conditions, being mostly built as questionnaires made of items that seek to measure - through organized response in the form of numerical scales - how aspects of people's lives are affected by poor health. However, it is emphasized that the quality of life is a construct, which cannot be fully operationalized and directly measured, i.e., the numerical measures are considered imperfect indexes^(^ 5 ^)^.

The interest in the use, creation, and validation of tools for quality of life in the field of child health has gradually developed in the Brazilian scientific community. This may result from discussions about the importance of the inclusion of instruments dealing with the assessment of quality of life in routine health care services. Nevertheless, we observed a shortage of national instruments, which appears to reflect the difficulty of the scientific community to develop tools for assessing quality of life that apply to the socio-cultural diversity of the country^(^ 12 ^)^.

**Chart 1:**
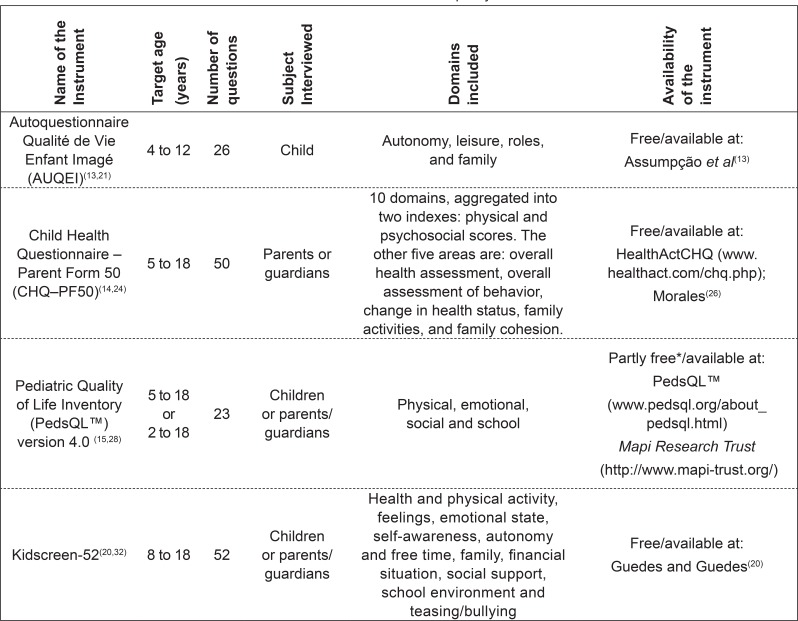
Characteristics of validated instruments in Brazil to assess the quality of life of children *Gratuity depends on the purpose for which the instrument is used. More information about potential utilization rates can be obtained at Mapi Research Trust (http://www.mapi-trust.org/)

Over the years, instruments were created to assess quality of life, such as the WHO Instrument for Assessment of Quality of Life (WHOQOL-100)^(18) ^and the 12-Item Short-Form Health Survey (SF-12)^(^ 19 ^)^. To further specify the assessment of quality of life, instruments were developed for specific population groups such as children. Among the instruments specifically designed to assess the quality of life of children, validated for the Brazilian language and culture, we highlight the *Autoquestionnaire Qualité de Vie Enfant Imagé* (AUQEI)^(^ 13 ^)^, the Child Health Questionnaire - Parent Form 50 (CHQ-PF50)^(^ 14 ^)^, the Pediatric Quality of Life Inventory (PedsQL(tm)) version 4.0^(15) ^and the Kidscreen-52^(^ 20 ^)^, which are the instruments considered in the present review (Chart 1).

## Autoquestionnaire Qualité de Vie Enfant Imagé

The AUQEI instrument is a quality of life scale developed by Manificat et al^(^ 21 ^)^, translated and validated into Brazilian culture and language in children from 4 to 12 years old^(^ 13 ^)^. This instrument is intended to assess the subjective feeling of well-being, assuming that the individuals under development are and have always been able to express themselves about their subjectivity. The questionnaire contains 26 questions covering the domains: autonomy, leisure, and family roles. To facilitate the application and understanding, the questionnaire uses pictures of faces expressing different emotional states (very unhappy, unhappy, happy, very happy), and the child himself/herself answers each question by choosing one of four answer options. Therefore, without a time limit, the child indicates the answer (face) that best matches his/her feeling against the proposed domain. Prior to the application of the questions, the child is asked to express his/her feelings to each of the response options. For example, for the answer that represents "very happy," the child is induced to remember a previous situation in which he/she had such feeling, in order to better understand the response options^(^ 13 ^)^.

To validate this tool for Brazilian language, we assessed 353 schoolchildren aged 4-12 years in the municipality of São Paulo, state of São Paulo, establishing a cutoff of 48 for the general population; values below this score were considered unsatisfactory quality of life. The study had internal validity, with Cronbach coefficient of 0.71, indicating adequate reliability of the instrument^(^ 13 ^)^.

The AUQEI has been widely used in studies that seek to evaluate the quality of life in healthy children and in children with some illness. One study showed similar quality-of-life indexes in 20 children aged 4-12 years old with autism, compared to 20 normal children matched for sex and age^(^ 22 ^)^.

Barreire et al^(23) ^evaluated the quality of life of 20 ostomized children attending a public reference service in São Paulo. In this study, we used the AUQEI questionnaire translated and adapted to Brazilian culture, applied to children and their mothers, obtaining, respectively, total mean scores of 51.95±7.90 and 49.60±5.60, which corresponded to the satisfactory quality of life in the perception of mothers and children^(^ 23 ^)^.

The AUQEI is easy to administer, has a relatively low number of questions, is self-administered, and without time limit, since children need to reflect on their feelings for each domain^(^ 13 ^)^. This questionnaire has low complexity because it uses faces that express feelings, being, therefore, easy to understand for children. 

To purchase the instrument in its entirety, one should access the study of Assumpção et al^(^ 13 ^)^, who conducted its validation for Brazil and presented the complete instrument in their study. We emphasize the importance of respecting the copyrights of the work, properly citing the authors who developed and validated the instrument.

## Child Health Questionnaire - Parent Form 50

The CHQ-PF50 is a generic instrument that assesses health-related quality of life in children^(^ 24 ^)^, which was adapted for the Brazilian culture by Machado et al^(^ 14 ^)^ in children with juvenile idiopathic arthritis and in healthy children, obtaining a Cronbach's coefficient of 0.7.

The CHQ-PF50 questionnaire consists of 50 questions designed to assess the physical and psychosocial well-being of children and adolescents aged from 5 to 18 years, and was responded by parents or guardians. The construct has 15 fields, each with a score on a scale of zero to 100; the higher the score, the better the state of health, satisfaction, and well-being. Among these domains, ten are aggregated into two indexes, the physical and psychosocial scores with a score from zero to 50 for each score. The ten aggregated domains are: physical functioning, social role of the limitation of daily activities due to emotional and behavioral aspects, social role of the limitation of daily activities due to physical capacity, body pain or discomfort, behavior, mental health, self-esteem, perceptions of health, emotional impact on the family, and impact on parents' time. The other five domains are: overall health assessment, global assessment of behavior, change in health status, family activities, and family cohesion^(^ 25 ^,^ 26 ^)^.

The questionnaire is self-administered to parents or guardians, who were instructed to base their information on the experiences lived by the child in the past four weeks, except in the scale on the overall health status, which refers to the last 12 months. To calculate each score, at least 50% of items answered in each scale are required^(^ 26 ^)^.

Faleiros and Machado^(^ 25 ^)^, in a study to assess health-related quality of life in 100 children with functional defecation disorders, used the CHQ-PF50, comparing their results with those of healthy children from a previous study that validated the instrument for Brazilian culture. In this study, the authors showed that the values ​​of the physical and psychosocial scores, as well as other areas of the instrument, were lower in children with disorders compared to healthy children.

A study used the CHQ-PF50 to assess the impact of allergic rhinitis on health-related quality of life in children and adolescents. The questionnaire was completed by parents or caregivers of 23 children and adolescents and showed that the scores obtained by the patients were lower (*p*<0.05) than those from the healthy control group in physical and psychosocial indexes and in most areas, showing that allergic rhinitis appears to negatively impact on quality of life^(^ 27 ^)^. 

The CHQ-PF50 has a relatively large number of questions (50 questions), which may require a long time for application. On the other hand, it is a self-administered instrument to parents, facilitating its use. A previous study highlighted the ease in implementation, considering the clarity of the version translated into Portuguese^(^ 25 ^)^. 

More information about the instrument and which languages ​​it has been translated to, as well as one of its versions in full can be obtained on the website HealthActCHQ (http://www.healthact.com/chq.php). It is noteworthy that some studies, such as the one by Morales^(^ 26 ^)^, present the full version of the instrument in Portuguese, as well as guidelines for its application and analysis.

## Pediatric Quality of Life Inventory version 4.0

The PedsQL(tm) was developed to measure the health-related quality of life in children and adolescents from 5 to 18 years and a questionnaire for parents of children and adolescents between 2 and 18 years old, and it can be used in patients with chronic health disorders or in healthy children and adolescents^(^ 28 ^)^. 

This instrument was translated and validated for the Brazilian culture^(^ 15 ^)^ and has 23 items that address the following dimensions: physical (eight items), emotional (five items), social (five items), and school (five items), which are developed from discussion groups, cognitive interviews, and pretests^(^ 29 ^)^. The assessment of children includes the following age groups: 5-7, 8-12, and 13-18 years. The questionnaire of the parents includes age groups from 2-4 years (preschooler), 5-7 (young child), 8-12 (child), and 13-18 years (adolescent). The items for each of the questionnaires are similar, differing in language, that is appropriate to the level of development and the use of first or third person^(^ 15 ^)^. For its application, approximately five minutes are needed^(^ 29 ^)^. The questions ask the individual how much each item represented a problem in the last month, with a range of responses from five options (0 - it is never a problem; 1 - it is hardly ever a problem; 2 - it is occasionally a problem; 3 - it is often a problem; 4 - it is almost always a problem). Negative questions are scored inversely in a scale from 0-100 to (0-100; 1-75; 2-50; 3-25; 4-0); thus, the higher the score, the better the quality of life. The scores of the dimensions can be computed as the sum of the items divided by the number of items answered and, if more than 50% of the items of the dimensions are missing, the score is not computed^(^ 15 ^)^.

The use of the PedsQL was demonstrated in a previous study that aimed to evaluate the quality of life of 50 obese children compared to 81 normal weight, aged 8-12 years, showing that obese children had lower quality of life in all four areas compared to normal weight children, with a significant difference in the physical, emotional, social, psychosocial, and general quality of life domains. The median overall quality of life of obese children was 69.9, while that of normal weight was 82.2 on a scale of 100 points^(^ 30 ^)^.

Klatchoian et al^(^ 31 ^)^ investigated the impact of demographic, social, economic, and family quality of life of 240 school children between 2 and 18 years old from São Paulo, with the PedsQL applied orally. The authors found satisfactory quality-of-life scores between the investigated schools compared to other populations of urban children. Furthermore, there was significant difference in emotional, social, and psychosocial aspects, and in the total score, with higher scores in children from social classes A + B, followed by the class C and the D + E, in all situations.

An advantage of the PedsQL is presenting two versions, one for parents and one for children and adolescents^(^ 15 ^)^, which allows greater use according to the characteristics of the study to be performed. Originally, the tool was proposed to be self-administered^(^ 28 ^)^, but the validation study for the Brazilian culture demonstrated the ability to be administered by the interviewer^(^ 15 ^)^. We also emphasize that the application of the instrument was considered fast and easy as well as the calculation of summary scores and scales^(^ 15 ^)^. 

More information about the PedsQL, such as versions in full and copyrights may be obtained from the PedsQL(tm) website - Measurement Model for the Pediatric Quality of Life Inventory(tm) (http://www.pedsql.org/about_pedsql.html). Versions of the instrument and permission for use may be requested from the Mapi Research Trust (http://www.mapi-trust.org/). 

## Kidscreen-52

*Kidscreen-52* was developed in Europe^(^ 32 ^)^ and validated in Brazil^(^ 20 ^)^ to assess and monitor the quality of life in children and adolescents.w

The instrument has 52 questions divided in the following dimensions: health and physical activity, feelings, emotional state, self-awareness, autonomy and free time, family/home environment, financial aspect, friends and social support, school environment, and teasing/bullying. This questionnaire provides practical steps for clinicians and researchers to assess the well-being and the subjective health of both healthy children and adolescents and individuals with chronic disease. The answers of the questions are distributed on a Likert scale of one to five points and refer to events that occurred in the week previous to the interview. It is also noteworthy the possibility of estimating the overall quality of life of the instrument. It is notable that the questionnaire has a version for parents/guardians, evaluating the same components of the version for children/teenagers^(^ 20 ^)^. Translation and validation of this instrument to Brazil occurred in a sample of 758 students of both sexes and 653 parents/guardians, obtaining satisfactory Cronbach values ​​between 0.725 and 0.894^(^ 20 ^)^.

This instrument is self-administered and can take 30 minutes on average^(^ 20 ^)^. The full version can be found in the study by Guedes and Guedes^(^ 20 ^)^, who conducted their validation in Brazil.

## General Comments

The questionnaires developed to measure the quality of life of children should be answered by themselves, because they have the right to express their opinions and to have them respected^(^ 5 ^)^. However, when very young, children may have trouble understanding the instrument. Furthermore, some may be disabled, presenting difficulty to provide information on their quality of life, being necessary that the questionnaire be answered by parents or guardians^(^ 5 ^)^. It is noteworthy that the PedsQL and the Kidscreen-52 have versions for children and another for parents or guardians, allowing the choice according to the design and the sample to be investigated.

As for the time spent to administer the questionnaire, it is believed that the smaller the number of questions, the shorter time may be required. However, the low number of questions does not mean that the instrument is easy to understand. Among those presented in this study, PedsQL and AUQEI have fewer questions. It is noteworthy that the AUQEI has a response system in the form of drawings that express feelings^(^ 13 ^)^, which may facilitate their understanding. The four instruments are self-administered, but the PedsQL can be administered by the interviewer^(^ 15 ^)^.

The validation process of some of the presented instruments was performed on specific samples chosen by researchers. However, due to the multicultural characteristics of Brazil, it is likely that specific cultural traits influence the understanding of some item of the questionnaire^(^ 20 ^)^. It is noteworthy that due to the low socioeconomic status of specific groups, the instruments may require adjustment in its application^(^ 15 ^)^.

The health-related indicators of quality of life assess the quality of life related to health conditions, emphasizing functional disorders and disabilities that can affect people^(^ 33 ^)^. In this context, instruments were also created to evaluate the quality of life related to the specific conditions of health and disease, such as, for instance, oral health^(^ 34 ^)^, asthma^(^ 35 ^)^ and HIV^(^ 36 ^)^, which were not presented because they were outside the scope of this review.

It becomes evident that the use of the instruments discussed in this study should be done cautiously, respecting the copyrights. In general, validation studies, as well as those that originated the instruments do not have clear information regarding copyrights. However, it is extremely important to reference and cite properly the people responsible for the creation and validation of instruments. We also emphasize that some instruments do not include all childhood age groups; Furthermore, due to the cultural diversity of Brazil, some adjustments to the instruments or even the validation of new instruments on these cultural specificities seem to be necessary. We also highlight the importance of some extremely important domains in the current context of childhood, such as bullying, which was present only in the Kidscreen-*52* ^(^ 20 ^)^.

## Conclusions

The evaluation of quality of life is essential due to its multidimensional character, encompassing social, psychological, and health issues of individuals. Therefore, among the age groups to be evaluated, the children stand out. The instruments described in this study assess the child's own perception of his/her quality of life, although some are also applied to parents. There have been a growing number of studies that seek to assess children's quality of life. Nevertheless, due to the cultural diversity of Brazil, besides the fact that some instruments do not include all childhood age groups or do not have the domains that are currently relevant, such as bullying, we recommend the creation or validation of new instruments to be widely used in the country.
