# Erratum

**DOI:** 10.1016/j.rpped.2014.12.002

**Published:** 2015-03

João Gabriel S. Souza^a,^^*^, Marcela Antunes Pamponet ^a^, Tamirys Caroline S. Souza^b^, Alessandra Ribeiro Pereira^c^, Andrey George S. Souza^c^, Andréa Maria E. de B. L. Martins^c^

^a^ Funorte, Montes Claros, MG, Brazil

^b^ Universidade de Aquino Bolivia (UDABOL), Santa Cruz de La Sierra, Bolivia

^c^ Universidade Estadual de Montes Claros (UNIMONTES), Montes Claros, MG, Brazil

**In the review article "Tools used for evaluation of Brazilian children's quality of life"** [Rev Paul Pediatr 2014;32(2):272-8], on page 274, table 1, sixth column ("Availability of the instrument"), on the second line of the table, where it reads:

"Free/available from: HealthActCHQ (www.healthact.com/chq.php); Morales^26^"

It should read:

"Permission needed. Exclusive intellectual property of HACHQ, protected by international copyright laws (http://www.healthactchq.com/)".

On page 276, first column, second paragraph, where it reads:

"More information about the instrument and which languages it has been translated to, as well as one of its versions in full can be obtained on the website HealthActCHQ (http://www.healthact.com/chq.php). It is noteworthy that some studies, such as the one by Morales,^26^ present the full version of the instrument in Portuguese, as well as guidelines for its application and analysis."

It should read:

"More information about the instrument and which languages it has been translated to can be obtained via the HealthActCHQ portal (http://www.healthactchq.com/). It is noteworthy that the instrument is of exclusive intellectual property of HealthActCHQ e and is totally protected by USA and international copyright laws. CHQ cannot be used without permission from HACHQ".

