# Supplementary material for: Tools used for evaluation of Brazilian children's quality of life
Source: Rev Paul Pediatr. 2014 Jun;32(2):272–8. doi: 10.1590/0103-0582201432214313 (PMC4183021; doi:10.1590/0103-0582201432214313)
Supplement: Translation

# Translation: Instrumentos utilizados na avaliação da qualidade de vida de crianças brasileiras

**Authors:** João Gabriel S. Souza, Marcela Antunes Pamponet, Tamirys Caroline S. Souza, Alessandra Ribeiro Pereira, Andrey George S. Souza, Andréa Maria E. de B. L. Martins

**Affiliations:** 1 Funorte, Montes Claros, MG, Brasil; 2 Universidade de Aquino Bolivia (Udabol), Santa Cruz de La Sierra, Bolívia; 3 Universidade Estadual de Montes Claros (Unimontes), Montes Claros, MG, Brasil

**Keywords:** qualidade de vida, criança, questionários

## Abstract

**OBJETIVO:**

Realizar uma revisão da literatura sobre instrumentos utilizados na avaliação da qualidade de vida de crianças, validados para o português e para a cultura brasileira.

**FONTES DE DADOS:**

Pesquisaram-se artigos científicos nos portais Medline, Lilacs e SciELO, por meio da combinação dos descritores "qualidade de vida", "criança" e "questionários", além de sua versão em inglês.

**SÍNTESE DOS DADOS:**

Dentre os instrumentos criados para avaliar a qualidade de vida em crianças e que foram validados para o português e para a cultura brasileira, destacam-se o *Autoquestionnaire Qualité de Vie Enfant Imagé* (AUQEI), o *Child Health Questionnaire - Parent Form 50* (CHQ-PF50), o *Pediatric Quality of Life Inventory* (PedsQL(tm)) *version 4.0* e o *Kidscreen-52*. Ressalta-se que alguns instrumentos não contemplam todas as idades da infância ou não possuem domínios considerados relevantes atualmente no contexto da infância, como o *bullying*. Além disso, devido à diversidade cultural do Brasil, podem ser necessárias adaptações dos instrumentos existentes ou validação de outros.

**CONCLUSÕES:**

Constata-se a existência de instrumentos validados no Brasil passíveis de serem utilizados para aferir a qualidade de vida de crianças. No entanto, considera-se a necessidade de adaptar ou validar outros instrumentos internacionais para suprirem as deficiências existentes.

## Introdução

A Organização Mundial da Saúde (OMS) definiu qualidade de vida como "a percepção do indivíduo de sua posição na vida no contexto da cultura e sistema de valores nos quais ele vive e em relação aos seus objetivos, expectativas, padrões e preocupações"^(^ ^1^ ^)^. Tal definição ressalta o caráter multidimensional da qualidade de vida, que engloba diferentes aspectos, entre eles, físico, funcional, além do bem-estar psicológico e social^(^ ^2^ ^,^ ^3^ ^)^. Há um crescente reconhecimento de que a qualidade de vida refere-se a algo muito mais amplo do que a saúde^(^ ^4^ ^)^. A avaliação da qualidade de vida exige que os profissionais de saúde não analisem apenas questões biológicas relacionadas à doença, devendo-se considerar uma abordagem psicossocial. Consequentemente, na década de 1990, consolidou-se a ideia de que os instrumentos de mensuração da qualidade de vida devem considerar a perspectiva das pessoas e não se restringir à perspectiva de profissionais de saúde e pesquisadores^(^ ^5^ ^)^. Estudos prévios objetivaram avaliar a qualidade de vida, sendo, em sua maioria, voltados para adultos ou idosos^(^ ^6^ ^,^ ^7^ ^)^, salientando, no entanto, a necessidade de avaliação da qualidade de vida em crianças^(^ ^8^ ^,^ ^9^ ^)^.

A avaliação da qualidade de vida em crianças foi inicialmente realizada pela percepção dos pais, considerando-se que crianças seriam incapazes de realizar tal tarefa. No entanto, maiores esclarecimentos permitiram constatar a capacidade desse grupo populacional de avaliar e compreender questões relacionadas à sua vida^(^ ^10^ ^)^, ainda que os pais possam ter uma percepção diferente de seus filhos^(^ ^11^ ^)^. Portanto, a inclusão de crianças como sujeitos de pesquisa depende da sua capacidade, respeitando-se seus limites no processo complexo relacionado à qualidade de vida e à saúde. Considera-se também a habilidade e a criatividade do pesquisador para adaptar instrumentos que se tornem interessantes e mobilizem as crianças a socializarem suas experiências^(^ ^12^ ^)^. Dessa forma, desenvolveram-se alguns instrumentos com intuito e possibilidade de serem utilizados para avaliar a qualidade de vida em crianças^(^ ^13^ ^-^ ^15^ ^)^.

Nesse contexto, o objetivo do presente estudo foi realizar uma revisão da literatura sobre os instrumentos validados para o português e para a cultura brasileira a fim de avaliar a qualidade de vida em crianças. Pesquisaram-se artigos científicos nos portais Medline, Lilacs e SciELO, utilizando-se a combinação dos descritores "qualidade de vida", "criança" e "questionários", além de suas versões em inglês. Após a junção dos descritores e a aplicação de filtros existentes nas bases de dados, tais como estudos em crianças, estudos de validação e estudos realizados no Brasil, computaram-se 637 trabalhos científicos. A partir da leitura dos resumos, excluíram-se os trabalhos que não abordavam a validação de instrumentos e que tinham como objetivo avaliar a qualidade de vida relacionada a uma condição de saúde ou doença específica. Portanto, incluíram-se quatro estudos que realizaram a validação de instrumentos genéricos da qualidade de vida em crianças para o português e para a cultura brasileira. A busca bibliográfica não se limitou a um período específico.

## Instrumentos de qualidade de vida

Os questionários são instrumentos amplamente utilizados em investigações científicas e podem ser úteis, entre outros aspectos, para estimar a necessidade de tratamento, investigar os determinantes do processo saúde-doença, avaliar serviços de saúde^(^ ^16^ ^)^ e a qualidade de vida. A criação de instrumentos para avaliar qualidade de vida psicometricamente válidos apresenta dificuldade considerável devido à sua característica subjetiva, sendo influenciada por aspectos temporais e culturais. Além disso, a característica multidimensional da qualidade de vida que se relaciona com o meio ambiente em aspectos físicos, psicológicos, relações sociais e crenças pessoais deve ser considerada, levando-se em conta que o objeto da avaliação é a percepção do respondente/paciente^(^ ^17^ ^)^.

Em geral, os indicadores de qualidade de vida associam-se às condições de saúde, sendo, na maioria, construídos como questionários compostos de itens que procuram medir - por meio de respostas organizadas sob a forma de escalas numéricas - o quanto aspectos da vida das pessoas são afetados pelas condições de saúde. No entanto, ressalta-se que a qualidade de vida é um construto, não podendo ser completamente operacionalizado e diretamente medido, ou seja, as medidas numéricas são consideradas índices imperfeitos^(^ ^5^ ^)^.

O interesse na utilização, criação e validação de instrumentos de qualidade de vida no campo da saúde da criança tem se desenvolvido gradualmente na comunidade científica brasileira. Tal fato pode decorrer de discussões sobre a importância da inclusão de instrumentos que contemplem a avaliação da qualidade de vida nos serviços assistenciais rotineiramente. Apesar disso, constata-se a escassez de instrumentos nacionais, o que parece refletir a dificuldade da comunidade científica em desenvolver instrumentos de avaliação de qualidade de vida que se apliquem à diversidade sociocultural do país^(^ ^12^ ^)^.

Ao longo dos anos, construíram-se instrumentos para avaliar a qualidade de vida, como, por exemplo, o Instrumento de Avaliação da Qualidade de Vida da OMS (WHOQOL-100)^(18) ^e o *12*-*Item Short-Form Health Survey* (SF-12)^(^ ^19^ ^)^. Para especificar melhor a avaliação da qualidade de vida, criaram-se instrumentos para grupos populacionais específicos, como as crianças. Dentre os instrumentos criados especificamente para avaliar a qualidade de vida de crianças, validados para a língua e cultura brasileiras, destacam-se o *Autoquestionnaire Qualité de Vie Enfant Imagé* (AUQEI)^(^ ^13^ ^)^, o *Child Health Questionnaire - Parent Form 50* (CHQ-PF50)^(^ ^14^ ^)^, o *Pediatric Quality of Life Inventory* (PedsQL^(tm))^ *version 4.0**^(15) ^*e o *Kidscreen-52**^(^* ^20^ ^)^, sendo estes considerados na presente revisão (Quadro 1).

**Quadro 1:** Características dos instrumentos validados no Brasil para avaliar a qualidade de vida de crianças *A gratuidade depende do fim para o qual instrumento será utilizado. Mais informações sobre possíveis taxas de utilização podem ser obtidas no Mapi Research Trust (http://www.mapi-trust.org/)

## Autoquestionnaire Qualité de Vie Enfant Imagé

O instrumento AUQEI é uma escala de qualidade de vida desenvolvida por Manificat *et al**^(^* ^21^ ^)^, traduzido e validado para língua e cultura brasileiras em crianças de quatro a 12 anos^(^ ^13^ ^)^. Esse instrumento tem o intuito de avaliar a sensação subjetiva de bem-estar, partindo do pressuposto de que o indivíduo em desenvolvimento é, e sempre foi, capaz de se expressar quanto à sua subjetividade. O questionário possui 26 questões, abrangendo os domínios: autonomia, lazer, funções e família. Para facilitar a aplicação e a compreensão, o questionário utiliza imagens de faces que expressam diferentes estados emocionais (muito infeliz; infeliz; feliz; muito feliz), sendo que a própria criança responde cada questão escolhendo uma das quatro opções de respostas. Portanto, sem tempo definido, a criança indica a resposta (face) que melhor corresponde ao seu sentimento frente ao domínio proposto. Anteriormente à aplicação das questões, solicita-se à criança que expresse seus sentimentos a cada uma das opções de respostas. Por exemplo, para a resposta que representa "muito feliz", a criança é induzida a lembrar de alguma situação prévia em que ela teve tal sentimento, a fim de melhor compreender as opções de resposta^(^ ^13^ ^)^.

Para validar esse instrumento para língua brasileira, avaliaram-se 353 escolares de quatro a 12 anos de São Paulo, SP, estabelecendo-se para a população geral um ponto de corte de 48 e considerando-se qualidade de vida insatisfatória abaixo desse escore. O estudo apresentou validade interna, com coeficiente de Cronbach de 0,71, indicando confiabilidade adequada do instrumento^(^ ^13^ ^)^.

O AUQEI vem sendo amplamente utilizado em investigações que buscam avaliar a qualidade de vida em crianças saudáveis e com alguma doença. Um estudo mostrou índices de qualidade de vida semelhantes em 20 crianças de quatro a 12 anos com autismo, comparadas a 20 crianças normais, pareadas por sexo e idade^(^ ^22^ ^)^.

Barreire *et al**^(23) ^*avaliaram a qualidade de vida de 20 crianças ostomizadas atendidas em um serviço público de referência do município de São Paulo. Nesse estudo, utilizou-se o questionário AUQEI traduzido e adaptado para a cultura brasileira, aplicado às crianças e às suas mães, obtendo-se, respectivamente, escores médios totais de 51,95±7,90 e 49,60±5,60, que corresponderam à qualidade de vida satisfatória na percepção de mães e crianças^(^ ^23^ ^)^.

O AUQEI é um instrumento de fácil aplicação, possui um número relativamente baixo de questões, é autoaplicável e com tempo indeterminado de aplicação, já que a criança precisa refletir sobre seu sentimento em relação a cada domínio^(^ ^13^ ^)^. Esse questionário possui baixa complexidade por utilizar faces que expressam sentimento, sendo, assim, de fácil entendimento para crianças. 

Para adquirir o instrumento na íntegra, pode-se acessar o estudo de Assumpção *et al**^(^* ^13^ ^)^, que realizaram a sua validação para o Brasil e apresentaram o instrumento completo no seu trabalho. Ressalta-se a importância de respeitar os direitos autorais do trabalho, devendo-se referenciar corretamente aqueles que desenvolverem e validaram o instrumento.

## Child Health Questionnaire - Parent Form 50

O CHQ-PF50 é um instrumento genérico que avalia a qualidade de vida relacionada à saúde de crianças^(^ ^24^ ^)^, adaptado para a cultura brasileira por Machado *et al**^(^* ^14^ ^)^ em crianças com artrite idiopática juvenil e crianças saudáveis, obtendo-se coeficiente de Cronbach de 0,7.

O questionário CHQ-PF50 é composto de 50 questões, desenvolvido para avaliar o bem-estar físico e psicossocial de crianças e adolescentes de cinco a 18 anos de idade, sendo respondido pelos pais ou responsáveis. O construto possui 15 domínios, cada um com pontuação em escala de zero a 100 e, quanto maior a pontuação, melhor o estado de saúde, o grau de satisfação e o bem-estar. Desses domínios, dez são agregados em dois índices, o escore físico e o psicossocial, com a pontuação de zero a 50 para cada escore. Os dez domínios agregados são: capacidade física, papel social da limitação das atividades diárias devido a aspectos emocionais e comportamentais, papel social da limitação das atividades diárias devido à capacidade física, dor corporal ou desconforto, comportamento, saúde mental, autoestima, percepção sobre o estado de saúde, impacto emocional na família e impacto no tempo dos pais. Os outros cinco domínios são: avaliação global da saúde, avaliação global do comportamento, mudança no estado de saúde, atividade familiar e coesão familiar^(^ ^25^ ^,^ ^26^ ^)^.

O questionário é autoaplicável aos pais ou responsáveis, sendo estes orientados a basearem suas informações nas experiências vividas pela criança nas últimas quatro semanas, exceto na escala sobre o estado global de saúde, que se refere aos últimos 12 meses. Para calcular cada escore, é necessário pelo menos 50% de itens respondidos em cada escala^(^ ^26^ ^)^.

Faleiros e Machado^(^ ^25^ ^)^, em estudo para avaliar a qualidade de vida relacionada à saúde em 100 crianças com distúrbios funcionais de defecação, utilizaram o CHQ-PF50, comparando seus resultados com os de crianças saudáveis de estudo prévio que validou o instrumento para a cultura brasileira. Nesse estudo, os autores mostraram que os valores dos escores físico e psicossocial, assim como os demais domínios do instrumento, foram inferiores em crianças com distúrbio comparadas às saudáveis.

Um estudo utilizou o CHQ-PF50 para avaliar o impacto da rinite alérgica na qualidade de vida relacionada à saúde em crianças e adolescentes. O questionário foi respondido por pais ou responsáveis de 23 crianças e adolescentes e evidenciou que os escores obtidos pelos pacientes foram menores (*p*<0,05) que os do grupo controle saudável nos índices físico e psicossocial e na maioria dos domínios, mostrando que a rinite alérgica parece causar impacto negativo na qualidade de vida^(^ ^27^ ^)^. 

O CHQ-PF50 possui um número relativamente grande de questões (50 questões), o que pode requerer um tempo longo para sua aplicação. Por outro lado, o instrumento é autoaplicável para pais, facilitando seu uso. Estudo prévio ressaltou a facilidade na sua aplicação, considerando a clareza da versão traduzida para a língua portuguesa^(^ ^25^ ^)^. 

Mais informações sobre o instrumento e para quais idiomas já foi traduzido, bem como uma de suas versões na íntegra, podem ser obtidas via internet pelo portal do HealthActCHQ (http://www.healthact.com/chq.php). Ressalta-se que alguns trabalhos, como o de Morales^(^ ^26^ ^)^, apresentam a versão integral do instrumento em Português, assim como orientações para aplicação e análise.

## Pediatric Quality of Life Inventory version 4.0

O PedsQL^(tm)^ foi desenvolvido para aferir a qualidade de vida relacionada à saúde em crianças e adolescentes de cinco a 18 anos e um questionário para os pais de crianças e adolescentes entre dois e 18 anos, sendo possível utilizá-lo em pacientes com distúrbios de saúde crônicos ou em crianças e adolescentes saudáveis^(^ ^28^ ^)^. 

Esse instrumento foi traduzido e validado para a cultura brasileira^(^ ^15^ ^)^ e possui 23 itens que abordam as seguintes dimensões: física (8 itens), emocional (5 itens), social (5 itens) e escolar (5 itens), sendo estas desenvolvidas a partir de grupos de discussão, entrevistas cognitivas e pré-testes^(^ ^29^ ^)^. A avaliação das crianças inclui as seguintes faixas etárias: cinco a sete, oito a 12 e 13 a 18 anos. O questionário dos pais inclui as faixas etárias de dois a quatro anos (pré-escolar), cinco a sete (criança pequena), oito a 12 (criança) e 13 a 18 anos (adolescente). Os itens para cada um dos questionários são similares, diferindo quanto à linguagem adequada ao nível de desenvolvimento e ao uso da primeira ou terceira pessoa^(^ ^15^ ^)^. Para sua aplicação, são necessários, aproximadamente, cinco minutos^(^ ^29^ ^)^. As questões indagam ao indivíduo o quanto cada item representou um problema no último mês, com uma escala de respostas de cinco opções (0 - nunca é um problema; 1 - quase nunca é um problema; 2 - algumas vezes é um problema; 3 - frequentemente é problema; 4 - quase sempre é um problema). Perguntas negativas são pontuadas inversamente em uma escala de 0-100 (0-100; 1-75; 2-50; 3-25; 4-0); assim, quanto maior o escore, melhor a qualidade de vida. Os escores das dimensões podem ser computados como a soma dos itens dividida pelo número de itens respondidos, sendo que, caso haja mais de 50% dos itens da dimensão ausentes, o escore da dimensão não é computado^(^ ^15^ ^)^.

Evidenciou-se a utilização do PedsQL em estudo prévio que objetivou avaliar a qualidade de vida de 50 crianças obesas comparadas a 81 eutróficas, na faixa etária de oito a 12 anos, mostrando que crianças obesas apresentaram qualidade de vida inferior em todos os quatro domínios em relação às crianças eutróficas, com diferença significante nos domínios físico, emocional, social, psicossocial e na qualidade de vida geral. A mediana da qualidade de vida geral das crianças obesas foi 69,9, enquanto que a das eutróficas foi 82,2, em uma escala de 100 pontos^(^ ^30^ ^)^.

Klatchoian *et al**^(^* ^31^ ^)^ investigaram o impacto de fatores demográficos, sociais, econômicos e familiares na qualidade de vida de 240 escolares entre dois e 18 anos da cidade de São Paulo, com o PedsQL aplicado verbalmente. Os autores identificaram escores satisfatórios de qualidade de vida entre os escolares investigados, quando comparados a outras populações de crianças urbanas. Além disso, houve diferença significante nos aspectos emocional, social e no escore psicossocial e total, com escores mais elevados nas crianças das classes sociais A+B, seguidas pela classe C e pela D+E, em todas as situações.

Uma vantagem do PedsQL é apresentar duas versões, uma para os pais e outra para a criança ou adolescente^(^ ^15^ ^)^, o que permite maior possibilidade de utilização segundo as características do estudo a ser realizado. Originalmente, o instrumento foi proposto para ser autoaplicável^(^ ^28^ ^)^, porém o estudo de validação para a cultura brasileira demonstrou a possibilidade de ser administrado pelo entrevistador^(^ ^15^ ^)^. Ressalta-se também que a aplicação do instrumento foi considerada rápida e fácil, assim como o cálculo dos escores sumários e das escalas^(^ ^15^ ^)^. 

Mais informações sobre o PedsQL, como versões na íntegra e direitos autorais, podem ser obtidas no portal *on-line *do *The PedsQL**^(tm)^* *- Measurement Model for the Pediatric Quality of Life Inventory**^(tm)^* (http://www.pedsql.org/about_pedsql.html). Versões do instrumento e autorização para a utilização podem ser solicitadas ao *Mapi Research Trust* (http://www.mapi-trust.org/).

## Kidscreen-52

O *Kidscreen-52* foi desenvolvido na Europa^(^ ^32^ ^)^ e validado no Brasil^(^ ^20^ ^)^ para avaliar e monitorar a qualidade de vida de crianças e adolescentes.

O instrumento possui 52 questões distribuídas nas dimensões: saúde e atividade física, sentimentos, estado emocional, autopercepção, autonomia e tempo livre, família/ambiente familiar, aspecto financeiro, amigos e apoio social, ambiente escolar e provocação/*bullying*. Esse questionário fornece medidas práticas para clínicos e pesquisadores avaliarem o bem-estar e a saúde subjetiva de crianças e adolescentes tanto saudáveis quanto portadores de doença crônica. As respostas das questões são distribuídas em escala Likert de um a cinco pontos e fazem referência aos acontecimentos ocorridos na semana anterior à aplicação do questionário. Ressalta-se também a possibilidade de estimar a qualidade de vida global do instrumento. Destaca-se que o questionário possui uma versão para pais/tutores, avaliando-se os mesmos componentes da versão para crianças/adolescentes^(^ ^20^ ^)^. A tradução e a validação desse instrumento para o Brasil ocorreu em uma amostra de 758 escolares de ambos os sexos e 653 pais/tutores, obtendo-se valores satisfatórios de Cronbach entre 0,725 e 0,894^(^ ^20^ ^)^.

Esse instrumento é autoaplicável, podendo levar em média 30 minutos^(^ ^20^ ^)^. A versão completa do instrumento pode ser consultada no estudo de Guedes e Guedes^(^ ^20^ ^)^, que realizaram sua validação para o Brasil.

## Comentários gerais

Os questionários desenvolvidos para mensurar a qualidade de vida de crianças devem ser respondidos pelas próprias, pois elas têm o direito de expressar suas opiniões e de tê-las respeitadas^(^ ^5^ ^)^. No entanto, quando muito jovens, podem ter dificuldade para entender o instrumento. Além disso, algumas podem estar incapacitadas, apresentando dificuldade para fornecer informações sobre a sua qualidade de vida, sendo necessário que o questionário seja respondido pelos pais ou responsáveis^(^ ^5^ ^)^. Vale ressaltar que o PedsQL e o *Kidscreen-52* possuem versões para crianças e outra para pais ou responsáveis, o que permite a escolha conforme o delineamento e a amostra a ser investigada.

Quanto ao tempo gasto para aplicar o questionário, acredita-se que, quanto menor o número de questões, menor pode ser o tempo necessário. Entretanto, o número baixo de questões não significa que o instrumento é de fácil entendimento. Entre os apresentados neste estudo, o AUQEI e o PedsQL possuem menor número de questões. Ressalta-se que o AUQEI possui um sistema de resposta em forma de desenhos que expressam sentimentos^(^ ^13^ ^)^, o que pode facilitar seu entendimento. Os quatro instrumentos apresentados são autoaplicáveis, porém o PedsQL pode ser administrado pelo entrevistador^(^ ^15^ ^)^.

O processo de validação de alguns dos instrumentos apresentados foi realizado em amostras específicas de escolha dos pesquisadores. No entanto, devido à característica multicultural do Brasil, existe a possibilidade de que traços culturais específicos influenciem na compreensão de algum item do questionário^(^ ^20^ ^)^. Ressalta-se ainda que, devido ao baixo nível socioeconômico de grupos específicos, os instrumentos podem requerer adaptação na sua aplicação^(^ ^15^ ^)^.

Os indicadores de qualidade de vida associada à saúde avaliam a qualidade de vida relacionada às condições de saúde, enfatizando alterações funcionais e incapacidades que podem afligir as pessoas^(^ ^33^ ^)^. Nesse contexto, criaram-se também instrumentos para avaliar a qualidade de vida relacionada às condições específicas de saúde e doença, como, por exemplo, saúde bucal^(^ ^34^ ^)^, asma^(^ ^35^ ^)^ e HIV/AIDS^(^ ^36^ ^)^, que não foram apresentadas por estarem fora do escopo desta revisão.

Evidencia-se que a utilização dos instrumentos abordados no presente estudo deve ser feita cautelosamente, respeitando-se os direitos autorais. Em geral, os estudos de validação, assim como os que originaram os instrumentos, não apresentam informações claras sobre direitos autorais. Todavia, é de suma importância referenciar corretamente e citar os responsáveis pela validação e criação dos instrumentos ao utilizá-los. Ressalta-se também que alguns instrumentos não contemplam todas as idades da infância; além disso, devido à diversidade cultural do Brasil, parecem ser necessárias algumas adaptações dos instrumentos ou até mesmo a validação de novos instrumentos diante dessas especificidades culturais. Salienta-se também a importância de alguns domínios específicos no contexto atual da infância, como o *bullying*, presente apenas no *Kidscreen-52**^(^* ^20^ ^)^.

## Conclusões

A avaliação da qualidade de vida torna-se essencial devido ao seu caráter multidimensional, englobando questões sociais, psicológicas e de saúde dos indivíduos. Portanto, dentre os grupos etários a serem avaliados, destacam-se as crianças. Os instrumentos descritos neste estudo avaliam a percepção da própria criança sobre sua qualidade de vida, apesar de alguns serem aplicados também aos pais. Nota-se um crescimento no número de investigações que buscam aferir a qualidade de vida de crianças. Apesar disso, devido à diversidade cultural do Brasil, além do fato de alguns instrumentos relatados não contemplarem todas as idades da infância ou não possuírem domínios relevantes atualmente, como o *bullying*, considera-se a necessidade de criar ou validar novos instrumentos para serem amplamente utilizados no país.
