# Supplementary material for: Erratum
Source: Rev Paul Pediatr. 2015 Mar;33(1):130. doi: 10.1016/j.rpped.2014.12.002 (PMC4436965; doi:10.1016/j.rpped.2014.12.002)
Supplement: Translation

# Translation: Errata

João Gabriel S. Souza^a,^^*^, Marcela Antunes Pamponet ^a^, Tamirys Caroline S. Souza^b^, Alessandra Ribeiro Pereira^c^, Andrey George S. Souza^c^, Andréa Maria E. de B. L. Martins^c^

^a^ Funorte, Montes Claros, MG, Brasil

^b^ Universidade de Aquino Bolivia (UDABOL), Santa Cruz de La Sierra, Bolívia

^c^ Universidade Estadual de Montes Claros (UNIMONTES), Montes Claros, MG, Brasil

No artigo de revisão "Instrumentos utilizados na avaliação da qualidade de vida de crianças brasileiras"Rev Paul Pediatr 2014;32(2):272-8>, na p. 274, tabela 1, sexta coluna ("Disponibilidade do instrumento" na segunda linha da tabela, onde se lê: 

 "Gratuito/disponível em: HealthActCHQ (www.healthact.com/chq.php); Morales^26^" 

Leia-se: 

 "Necessita de permissão. Propriedade intelectual exclusiva da HACHQ e protegido pelas leis internacionais de copyright (http://www.healthactchq.com/)"

Na p. 276, primeira coluna, segundo parágrafo, onde se lê: 

 "Mais informações sobre o instrumento e para quais idiomas já foi traduzido, bem como uma de suas versões na íntegra, podem ser obtidas via internet pelo portal do HealthActCHQ (http://www.healthact.com/chq.php). Ressalta-se que alguns trabalhos, como o de Morales,^26^ apresentam a versão integral do instrumento em português, assim como orientações para aplicação e análise." 

Leia-se: 

"Mais informações sobre o instrumento e para quais idiomas já foi traduzido podem ser obtidas via internet pelo portal do HealthActCHQ (http://www.healthactchq.com/). Ressalta-se que o instrumento é de propriedade intelectual exclusiva da HealthActCHQ e foi totalmente protegido pelos EUA e pelas leis internacionais de copyright. O CHQ não pode ser usado sem a permissão do HACHQ."
